# Measurement of plantar pressure differences in the contralateral limb when using offloading modalities for diabetic foot ulcerations

**DOI:** 10.1002/jfa2.70028

**Published:** 2025-01-11

**Authors:** Ian Rong Yi Ngui, Jane Bowden, Sara L Jones, Rebecca Daebeler, Ryan S Causby

**Affiliations:** ^1^ The University of South Australia Allied Health & Human Performance Unit Adelaide SA Australia; ^2^ Flinders Medical Centre, Podiatry Department Bedford Park SA Australia

**Keywords:** diabetic foot, offloading, plantar pressure, podiatry

## Abstract

**Background:**

This study investigated the effect of various offloading devices commonly used for the management of diabetic foot ulcerations on peak plantar pressure and pressure–time integral of the contralateral limb.

**Methods:**

A quantitative, randomised and within‐subject repeated measures study was conducted in an outpatient gait laboratory. Outpatients with unilateral diabetic foot ulcers and adequate perfusion to the lower limb without an intrinsic limb‐length discrepancy who were able to walk were recruited for the study. They were also required to understand English. An in‐shoe pressure sensor was placed in the participants’ everyday shoes between their feet and insoles. Participants were asked to walk at their own speed and cadences with three stances recorded. Their peak and mean plantar pressures were recorded. This was repeated with four different offloading conditions: Darco APB™ All Purpose Boot, Darco APB™ All Purpose Boot with wool felt adhered to the bottom of the foot, DH Offloading Walker® and DH Offloading Walker® with Even‐Up™ on the contralateral foot.

**Results:**

The total sample comprised 22 adults (3 females and 19 males) aged between 34 and 78 years old (mean age, 57.6 ± 9.9 years). The results indicated that none of the regions of the foot showed a statistically significant difference in peak plantar pressure and pressure–time integral between the control condition and other offloading modalities, or between modalities.

**Conclusion:**

The use of offloading modalities for diabetic foot ulcers does not significantly affect peak plantar pressure or pressure–time integral measures on the contralateral limb. However, this should be considered with caution, as this population will possess the same risk factors in both the affected and the contralateral foot.

## 1 INTRODUCTION

It is well established that people living with diabetes are at a high risk of lower limb ulceration due to the onset of progressive peripheral neuropathy, as a consequence of persistent elevated blood glucose levels [1]. The subsequent absence of sensory input, together with associated biomechanical changes can lead to elevated plantar pressures and resultant tissue damage [2]. This may be the result of a peak in plantar pressure, or a more moderate increase in pressure over an extended period due to altered gait and plantar pressure distribution [2, 3]. Thus, a reduction in these pressures via offloading modalities should reduce or prevent tissue breakdown, the development of diabetic foot ulcers, and ultimately amputation.

The International Working Group on the Diabetic Foot guidelines recommend various offloading strategies for diabetic foot ulcers to decrease plantar pressures. Such offloading strategies may include the use of removable cast walkers (RCWs), post‐operative shoes, medical grade footwear and customised footwear [4]. To offload a diabetic foot ulcer, a nonremovable knee‐high offloading device is recommended as the first choice to offload the foot, followed by a removable knee‐high device and then a removable ankle‐high device [4]. Footwear and offloading devices combined with wool felt should be considered as the fourth‐choice treatment [4].

In clinical practice, it is common for patients to wear an offloading device on the affected foot, but they still wear their everyday shoe on the contralateral foot [5]. This could potentially lead to a height discrepancy or further affect biomechanics that may subsequently lead to an uneven pressure distribution on the contralateral foot.

To date, the investigation of plantar pressure changes on the foot in the presence of height or limb‐length discrepancies has been restricted to participants with a discrepancy as a consequence of wearing shoes of different thicknesses and walking barefoot [6, 7].

A previous study compared the effect of a simulated limb‐length discrepancy in participants with neuropathic foot ulcers by walking barefoot, wearing shoes with the same thickness and wearing shoes with a higher platform [6]. This study found an increase in peak plantar pressure in the foot with a relatively longer limb of 20 mm or more. However, this does not represent the scenario faced by patients requiring offloading in clinical practice.

Another study looked at the effect of height differences caused through the use of offloading modalities and found that there was improved comfort and gait mechanics when wearing an ankle‐high RCW and a contralateral limb lift [7]. The study only involved participants ‘at‐risk’ of developing diabetic foot ulcers, rather than active ulcers. There is a need for a study involving participants with active diabetic foot ulcers to ensure more valid findings and recommendations.

The aim of this study is to investigate the effect of various offloading devices commonly used for the management of diabetic foot ulcers on the peak and mean plantar pressure on the contralateral limb. We hypothesised that the contralateral foot would have an increase in peak plantar pressure and pressure–time integral compared to the foot with an active ulcer in a RCW, a post‐operative shoe or a post‐operative shoe with wool felt.

## 2 MATERIALS AND METHODS

This quantitative, randomised and within‐subject repeated measures study was conducted in the outpatient gait laboratory of Flinders Medical Center (Southern Adelaide Local Health Network), Adelaide, South Australia, from December 2021 to August 2022. Ethical approval was sought from the Southern Adelaide Clinical Human Research Ethics Committee prior to commencing the study.

Current patients receiving outpatient treatment within the Podiatry Department were recruited by clinicians. Participants were recruited if they were English‐speaking, aged between 18 and 80 years, with unilateral plantar foot ulcers caused by diabetes, had adequate perfusion to both limbs, had the ability to walk more than 500 m and with absence of a structural limb‐length difference. Potential participants were excluded if they have had a previous amputation on the contralateral limb. Potential participants were also excluded if they had a limb‐length difference of more than 1 mm. Vascular status or perfusion was assessed through palpation of dorsalis pedis and posterior tibial pulses. The ability to walk more than 500 m was self‐determined by participants prior to participation. An information sheet was provided prior, and consent was obtained in accordance with the Declaration of Helsinki.

Demographic characteristics (age, gender, height, weight and duration of diabetes) and limb‐length measures were collected. Limb‐length was measured from the anterior superior iliac spine to the medial malleolus in a standing position [8]. Participants had their foot ulcers covered with an OPSITE (Smith & Nephew, Watford, England) film dressing for protection and to prevent infection. Disposable nylon socks were provided to participants prior to plantar pressure measurement to ensure consistency between participants. Plantar pressure were measured using the F‐scan (Tekscan Inc, South Boston, Massachusetts) system, an in‐shoe pressure measuring system which has been shown to be valid and reliable [9]. Results from a previous study showed that the system demonstrated high repeatability (ICC = 0.86–0.94) [10]. The F‐scan (Tekscan Inc, South Boston, Massachusetts) was chosen over other systems as the insoles could be cut to the required size to accommodate varying footwear or offloading modalities.

A pair of F‐scan insoles was first trimmed to the required size, and then placed inside participants’ shoes. The F‐scan insoles were placed between the plantar surface (with socks on) and insole of their currently used footwear. Prior to pressure data collection, participants were directed to walk freely around the laboratory for 5 minutes to acclimatise to the equipment and pressure sensors to warm up the sensors to prevent creep [11]. The sensor insoles were then calibrated following manufacturer guidelines [11]. Subsequent to successful calibration, participants were requested to walk at their own speed and cadence along a 20‐m smooth walkway for a minimum of three stances on each foot. This was repeated with four different conditions; the order was randomised and predetermined using a computer‐generated randomisation table. Peak plantar pressure and pressure–time integral measurements were recorded for each of the offloading modalities, undertaken on the same day. The modalities were as follows:

1. Participants’ own current footwear on both feet (control)
2. Darco APB™ All Purpose Boot (Darco International, Huntington, WV, USA) on the foot with an active ulcer and their own current footwear on the contralateral foot
3. Darco APB™ All Purpose Boot on the foot with an active ulcer with wool felt adhered to the bottom of the foot around the ulcer and their own current footwear on the contralateral foot
4. DH Offloading Walker® (Össur, Reykjavik, Iceland) on the foot with an active ulcer and their own current footwear on the contralateral foot
5. DH Offloading Walker® on the foot with an active ulcer and their own current footwear with an Even‐Up™ on the contralateral foot

Participants were given a standardised rest period of 5 minutes between each testing condition to avoid fatigue and to allow sufficient time to change the offloading conditions. Qualified podiatrists were present for appropriate fitting of offloading modalities and application of wool felt around the ulcer site consistent with usual clinical practice. A double‐skived single layer of 10 mm wool felt was placed around the ulcer site with a cut‐out to offload the region.

**FIGURE 1:**
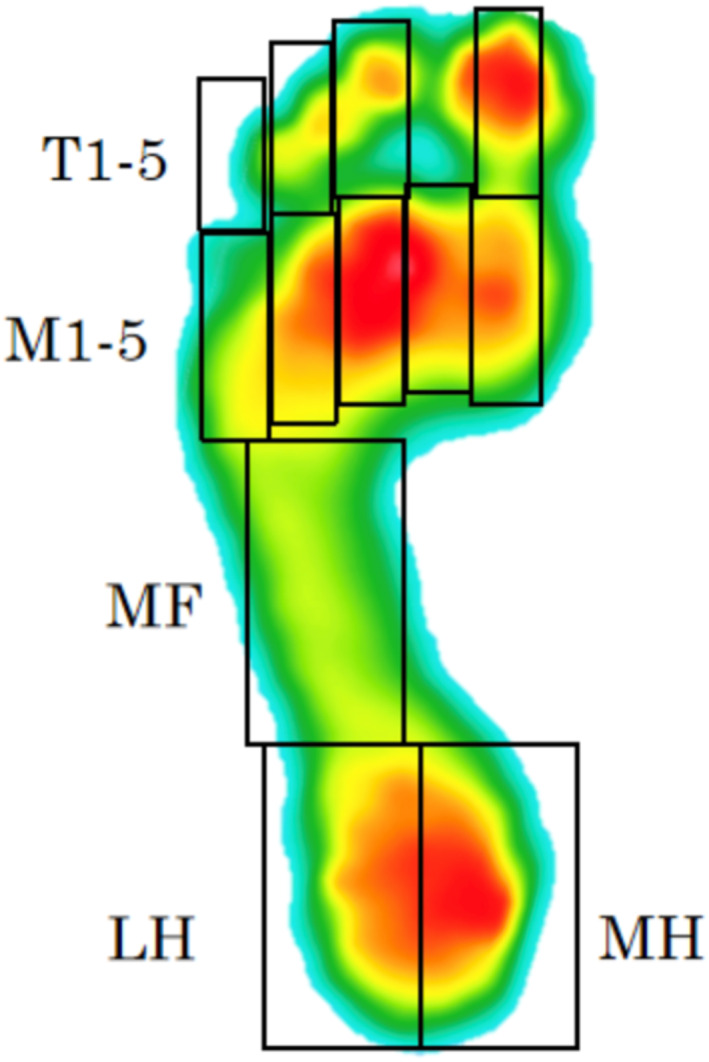
An example of the foot masking regions.

For data extraction, the first and last stance phases were excluded to minimise effects of acceleration and deceleration. The middle stance was masked into 12 separate regions using the auto‐masking feature within the F‐scan system [12]. The 12 regions were the first digit, second digit, third digit, fourth and fifth digits, first metatarsal, second metatarsal, third metatarsal, fourth metatarsal, fifth metatarsal, midfoot, medial heel and lateral heel. An example of the foot masking is shown below (see Figure 1).

Data from all participants were analysed using SPSS (version 28.0.0; SPSS Inc, Chicago Illinois). A linear mixed model was used to compare the different masked regions for each of the different conditions. Statistical significance was set at *p* < 0.05.

## 3 RESULTS

A total of 30 patients were invited to participate in the study, but 8 patients were excluded for not meeting the inclusion criteria. A total of 22 adults were recruited (3 females and 19 males) aged between 34 and 78 years (mean 57.6 ± 9.9 years). Table 1 outlines the participant demographics. A total of 22 participants enrolled for the study, but only 21 participants completed testing for all the modalities. An a priori power analysis indicated that 25 participants were needed to detect an effect size of 0.5 with 80% power.

**TABLE 1:** Demographics of the study population.

Demographics of study population	Number/Mean (SD)
Age (years)	57.6 (9.92)
Sex (F:M)	3:19
Height (cm)	179.2 (7.28)
Weight (kg)	100.0 (18.16)
Diabetes (type I: type 2)	4:18
Duration of diabetes (years)	17.6 (10.01)

As shown in Table 2, none of the regions analysed showed a statistically significant difference in peak plantar pressure between any of the offloading modalities and control (participants’ shoes). There was also no statistically significant difference between any of the offloading conditions (*p* = 0.990). Similarly, results showed that all regions of the foot failed to show a statistically significant difference between offloading conditions and control, or between conditions (*p* = 1.000) (Table 3).

**TABLE 2:** Peak plantar pressure in all regions of the contralateral foot in all modalities.

Regions	Peak pressure (N) Mean (SD)					*p*‐value
	Control (*n* = 22)	Post‐op shoe (*n* = 22)	Post‐op shoe with wool felt (*n* = 22)	RCW (*n* = 21)	RCW with even‐ Up™ (*n* = 21)	
Hallux	23.99 (14.55)	23.12 (15.82)	24.79 (16.68)	23.30 (15.32)	28.32 (24.42)	0.873
Second toe	18.06 (13.79)	18.09 (14.85)	17.16 (14.23)	19.19 (14.16)	20.14 (15.38)	0.969
Third toe	15.75 (11.98)	15.45 (10.88)	13.29 (9.56)	14.05 (9.84)	15.02 (11.38)	0.939
Fourth and fifth toe	10.12 (6.68)	10.82 (10.28)	9.15 (7.95)	9.31 (6.95)	8.52 (6.36)	0.888
First metatarsal	26.73 (12.94)	26.81 (11.81)	29.11 (16.67)	29.31 (16.35)	29.68 (17.46)	0.941
Second metatarsal	24.93 (18.07)	26.77 (20.91)	26.04 (21.24)	24.61 (19.85)	19.62 (12.94)	0.758
Third metatarsal	30.10 (19.26)	30.97 (20.78)	27.52 (18.55)	29.25 (27.75)	23.10 (25.14)	0.802
Fourth metatarsal	21.36 (12.72)	20.19 (11.52)	18.53 (10.62)	18.09 (10.78)	13.55 (7.22)	0.170
Fifth metatarsal	19.48 (13.23)	19.00 (13.95)	17.25 (11.28)	16.77 (11.47)	9.82 (4.73)	0.053
Midfoot	22.02 (14.09)	19.39 (10.79)	20.51 (12.76)	20.10 (14.00)	15.43 (8.97)	0.494
Medial heel	38.18 (16.47)	37.85 (17.99)	37.11 (16.00)	32.41 (16.96)	32.45 (14.69)	0.613
Lateral heel	29.96 (14.91)	30.80 (14.27)	30.46 (14.53)	26.72 (12.41)	23.64 (12.20)	0.376

*p < 0.05.

**p < 0.001.

*RCW = removable cast walker.

**TABLE 3:** Pressure–Time Integral in all regions of the contralateral foot in all modalities.

Regions	Pressure time integral (N/cm^2^) Mean (SD)					*p*‐value
	Control (*n* = 22)	Post‐op shoe (*n* = 22)	Post‐op shoe with wool felt (*n* = 22)	RCW (*n* = 21)	RCW with Even‐ Up™ (*n* = 21)	
Hallux	4.45 (3.27)	3.89 (2.50)	4.43 (3.17)	5.63 (5.45)	5.96 (5.02)	0.401
Second toe	4.83 (4.69)	4.45 (4.15)	4.42 (4.20)	5.82 (7.24)	5.63 (4.99)	0.843
Third toe	4.53 (3.34)	4.18 (2.97)	3.83 (2.64)	4.88 (3.71)	4.45 (3.10)	0.855
Fourth and fifth toe	3.07 (2.67)	2.97 (3.02)	2.77 (3.24)	3.13 (3.16)	2.86 (2.47)	0.994
First metatarsal	5.77 (3.26)	5.05 (2.71)	5.74 (3.99)	8.15 (9.28)	6.58 (4.24)	0.353
Second metatarsal	5.47 (3.54)	5.26 (3.57)	5.26 (4.09)	6.89 (6.63)	4.95 (2.70)	0.618
Third metatarsal	6.47 (3.68)	5.95 (3.42)	5.42 (2.95)	7.36 (5.31)	5.83 (5.44)	0.624
Fourth metatarsal	5.48 (2.95)	4.74 (2.64)	4.51 (2.53)	5.67 (3.81)	3.71 (2.05)	0.176
Fifth metatarsal	5.59 (2.81)	4.85 (2.75)	4.49 (2.44)	5.94 (5.31)	3.34 (2.18)	0.093
Midfoot	4.75 (1.76)	4.09 (1.46)	4.34 (2.13)	5.15 (4.28)	3.99 (1.85)	0.534
Medial heel	9.33 (3.84)	8.77 (5.37)	7.85 (3.81)	11.39 (15.59)	9.09 (4.43)	0.679
Lateral heel	6.59 (3.31)	5.78 (3.51)	5.09 (2.34)	7.77 (10.77)	5.25 (2.69)	0.486

*p < 0.05.

**p < 0.001.

*RCW = removable cast walker.

Data were not obtained for one participant for the modalities of DH Offloading Walker® and DH Offloading Walker® with an Even‐Up™ on the contralateral foot, due to the participant feeling unstable and reporting a lack of confidence to complete the study. Thus, measurements for the Darco APB™ All Purpose Boot and Darco APB™ All Purpose Boot with wool felt were included in the linear regression, but not the missing data.

## 4 DISCUSSION

This study aimed to study the effect of various offloading devices commonly used for the management of plantar foot ulcerations caused by diabetes on the peak and mean plantar pressure for the contralateral limb.

Based on previous literature, it was hypothesised that the contralateral foot would have an increase in peak plantar pressure and pressure–time integral when a post‐operative shoe with wool felt adhered to the foot of interest and a RCW was worn on the foot with an active ulcer [13]. However, our findings showed no significant differences on the contralateral foot between any of the experimental conditions.

Results from previous studies have shown that Even‐Up™ has been effective in reducing peak plantar pressure in multiple regions of the contralateral foot by reducing any potential height differences [14]. The results of this study showed the same trend in all of the regions of the foot, except for the hallux and first metatarsal, when participants were wearing a RCW with an Even‐Up™ on the contralateral foot. An unexpected finding was a higher peak plantar pressure and pressure–time integral on the hallux and first metatarsal region of the contralateral foot when wearing a RCW on the foot with an active diabetic foot ulcer and an Even‐Up™ on the contralateral foot, than the other conditions. While this difference was not significant, this was unexpected, as the purpose of an ‘even‐up’ is to ensure a more even distribution of pressure.

In patients with plantar foot ulcers caused by diabetes, a modality‐induced height discrepancy could significantly alter their gait. A healthy patient would most likely be able to accommodate the discrepancy caused by offloading devices through a combination of kinematic changes at different body levels, such as hip, knee or foot [14]. For example, the shorter leg could accommodate for limb‐length discrepancy by increasing the degree of plantarflexion and/or supination during stance and/or heel lift phases of gait. The longer leg could also adapt by increasing pronation and/or dorsiflexion of the midstance phase of gait [14].

However, diabetic peripheral neuropathy could alter the efficiency or ability of patients to alter their gait to accommodate changes caused by the height discrepancy from offloading modalities [1]. This is supported with a study by Sacco et al. [15] which found limited ankle joint mobility, the reduced recruitment of lower limb muscles and an increased stiffness in the soft tissues in people with diabetic peripheral neuropathy. Due to the reduction of the ankle joint motion, the heel lifts earlier after heel strike leading to an increased concentration of pressure on the midfoot and the forefoot due to alterations in normal foot rollover in gait and disruptions of the functional rockers of gait [16].

There are study limitations that must be considered. Firstly, the extent of neuropathy of participants was not considered. Previous studies have shown that people with greater loss of plantar cutaneous sensation due to diabetic peripheral neuropathy will have an altered gait pattern which could further affect plantar pressure distribution [17, 18, 19]. Further to this, neither the researchers nor participants were blinded during the data collection process. The present study did not look at different foot types among participants. A previous study showed that different foot types, similar to degree of neuropathy, affect plantar pressure distribution and peak plantar pressure in different regions of the foot [20]. Furthermore, the sample size of this study did not meet the required power analysis. In a study looking at the number of stances needed to obtain a valid and reliable in‐shoe plantar pressure data, three stances were shown to be suitable; however, for pressure–time integral, 12 stances were recommended [21]. In this study, a minimum of three stances were recorded and used for analysis. Future studies should consider a larger sample size with both practitioners and participants blinded and quantify the degree of neuropathy and different foot types of participants as well as increase the number of stance phases in each trial to improve the validity of the results. In this study, the placement of wool felt and fitting of offloading modalities were done by different qualified podiatrists based on availability on test days. Future studies should consider a single investigator to improve inter‐rater reliability. Future studies should also consider feedback from patients such as their comfort when walking as it is an essential component when making a decision on offloading modalities.

## 5 CONCLUSION

Based on the findings of this study, the use of offloading modalities for plantar foot ulcers caused by diabetes does not significantly increase peak plantar pressure or pressure–time integral on the contralateral limb. This suggests that these offloading modalities may not put the contralateral limb at an increased risk. However, the same risk factors for ulceration are likely present in the contralateral limb as in the affected foot, and these should always be considered when offloading an ulcer to prevent breakdown.

## AUTHOR CONTRIBUTIONS

**Ian Rong Yi Ngui**: Conceptualization; Data Curation; Investigation; Methodology; Formal Analysis; Writing ‐ Original Draft; Writing ‐ Review & Editing. **Ryan S Causby**: Conceptualization; Methodology; Writing ‐ Review & Editing. **Sara L Jones**: Writing ‐ Review & Editing. **Rebecca Daebeler**: Methodology; Investigation. **Jane Bowden**: Methodology; Investigation.

## CONFLICT OF INTEREST STATEMENT

The authors have declared no competing interests.

## ETHICS STATEMENT

Ethical approval was sought from the Southern%20Adelaide%20Clinical%20Human%20Research%20Ethics%20Committee prior to commencing the study.

## Data Availability

Data are available on request from the authors. The data that support the findings of this study are available from the corresponding author upon reasonable request.
